# SAPAP3, SPRED2, and obsessive-compulsive disorder: the search for fundamental phenotypes

**DOI:** 10.3389/fnmol.2023.1095455

**Published:** 2023-05-31

**Authors:** Ravi Philip Rajkumar

**Affiliations:** Department of Psychiatry, Jawaharlal Institute of Postgraduate Medical Education and Research (JIPMER), Puducherry, India

**Keywords:** obsessive-compulsive disorder, SAPAP3, SPRED2, animal models, endophenotypes, sensory over-sensitivity, neurodevelopmental model

## Introduction

Obsessive-compulsive disorder (OCD) is a psychiatric disorder characterized by recurrent unwanted thoughts (obsessions) and associated repetitive behaviors (compulsions), affecting around 1.1–1.3% of the global population (Stein et al., 2016a; Fawcett et al., 2020). Over the past two decades, researchers have identified several distinct domains or dimensions of OCD symptomatology, with apparently distinctive neural correlates and differential responses to specific treatments (Mataix-Cols et al., 1999, 2004, 2005; van den Heuvel et al., 2009; Kichuk et al., 2013; Williams et al., 2014). These dimensions have also been identified at a “sub-syndromal” level in up to 13% of a large sample of adults from six countries, and include Contamination/Cleaning, Harm/Checking, Symmetry/Ordering, Hoarding, Sexual/Religious, Somatic and Moral obsessions and compulsions (Fullana et al., 2010). These findings suggest that OCD is best understood not as a unitary disorder, but as a group of related disorders.

## Top-down and bottom-up models of OCD

Most contemporary models of OCD place a high degree of emphasis on the role of higher-level processes, such as cognitive flexibility or the sense of responsibility, in the genesis and maintenance of OCD symptoms. Such models can be considered as taking a “top-down” perspective (Poletti et al., 2022a). However, OCD-like phenomena have been documented in animals, such as dogs, cats and primates (Luescher et al., 1991; Overall and Dunham, 2002; Lutz, 2014). OCD is also frequently encountered in children, where higher-level cognitive processes are not yet fully developed (Geller, 2006). Such findings suggest a need for a “bottom-up” perspective based on evolutionary and neurodevelopmental models. Sigmund Freud was among the first to suggest a similarity between the rituals seen in OCD and behaviors that maintain social stability in the face of conflicting human drives. Though his model of OCD is no longer widely accepted, it does show some points of correspondence with contemporary biochemical or cognitive models (Katz, 1991; Moritz et al., 2011). More generally, it is now understood that many of the symptoms of OCD may represent exaggerations or distortions of phylogenetically ancient adaptive behaviors or defense mechanisms, whose purpose is to ensure individual or group wellbeing and safety (Boyer and Lienard, 2006; Feygin et al., 2006; Stein et al., 2016b). Such an evolutionary perspective regarding OCD entails a neurodevelopmental perspective, in which alterations in normal brain development could perturb basic, evolutionarily conserved neural processing systems and predispose to the development of OCD at specific stages of the life cycle in a “bottom-up” manner (Leckman and Bloch, 2008; Poletti et al., 2022a). Such mechanisms could potentially be identified in animals as well as humans.

## From SAPAP3 to SPRED2: OCD and neurodevelopment in rodents

In this connection, it is relevant to examine two particular rodent models of obsessive-compulsive disorder which share certain unexpected similarities. In 2009, it was observed that mice in whom the *SAPAP3* gene had been deleted exhibited compulsive behaviors and increased anxiety reminiscent of OCD (Welch et al., 2007). This gene codes for a protein that is highly expressed in the corpus striatum and involved in post-synaptic scaffolding, and its disruption was associated with altered glutamatergic, gamma-amino butyric-acid (GABA)-ergic and dopaminergic transmission in the orbitofrontal cortex, corpus striatum and nucleus accumbens. These changes were associated not just with OCD-like behavior, but with impairments in lower-level (sensory processing) and higher-level (reversal learning, a measure of cognitive flexibility) processes (Wan et al., 2011; Manning et al., 2021; Yang et al., 2021). Observation of neonatal mice deficient in *SAPAP3* has identified increases in ultrasonic vocalizations, a marker of altered communication and social development (Tesdahl et al., 2017).

More recently, it has been observed that mice in whom the *SPRED2* gene was knocked out exhibit OCD-like behavior and anxiety, both of which are highly similar to those observed in *SAPAP3-*deficient mice (Ullrich et al., 2018). *SPRED2* codes for a protein that is a key regulator of the Ras/ERK-MAPK pathway, an intracellular cascade that can be activated by brain-derived neurotrophic factor (BDNF); it has been shown to play a key role in neurogenesis and neural development, and possibly in synaptic vesicle transport. In *SPRED2*-deficient mice, alterations in neural transmission were observed in thalamo-amygdala circuits. Subsequently, it was found that these mice, like those in which *SAPAP3* had been deleted, also showed altered ultrasonic vocalizations. These changes were observed in both young and older mice, and appeared to increase with age (Hepbasli et al., 2021). That these changes reflect a developmental anomaly is supported by evidence that *SPRED2* is involved in central nervous system development in mice (Tuduce et al., 2010).

A relevant question in this context is whether alterations in either *SPRED2* or *SAPAP3* are associated with OCD in humans. While no studies of *SPRED2* in patients with OCD have been published to date, a cautious affirmative answer can be offered in the case of *SAPAP3*. A specific four-locus haplotype of *SAPAP3* has been associated with an earlier age of onset in OCD, again pointing to a possible effect on neurodevelopment (Boardman et al., 2011); an allelic variant in a specific single nucleotide polymorphism (*rs*6662980) of *SAPAP3* has been specifically associated with the Contamination/Washing dimension of OCD, as well as with a poor response to serotonin reuptake inhibitors (Naaz et al., 2020); and two single-nucleotide polymorphisms in *SAPAP3* have been associated with symptom severity in early-onset OCD (Mas et al., 2016). In addition, a genome-wide association study has found that variations in *SAPAP1* (also known as *DLGAP1*), coding for a protein related to *SAPAP3* which is also involved in synaptic connectivity, were significantly associated with clinical OCD (Stewart et al., 2013).

Copy number variants in *SAPAP1* and the related gene *SAPAP2 (DLGAP2)* have also been associated with childhood OCD (Gazzellone et al., 2016). Mice in which *SAPAP1* has been knocked out exhibit impaired scaffolding at glutamatergic synapses and altered social behavior (Coba et al., 2018). There is also evidence that variations in the *BDNF* and *NTRK2* genes, which are proximal components of the same cellular cascade as *SPRED2*, may exert a protective effect against OCD; these effects may be mediated by beneficial effects on this particular signaling pathway (Alonso et al., 2008).

Taken together, these findings suggest that genes involved in neurodevelopment and synaptic connectivity, when disrupted, induce not just OCD-like behavior but alterations in brain development, sensory processing, cognitive functioning and social behavior in animals. At least one of these genes is also associated with certain facets of OCD in humans. There is evidence from animal research that alterations in these genes are associated with functional changes involving prefrontal, striatal and limbic brain regions. The consistency of these findings across rodents and humans suggests that at least some of the genetic mechanisms underlying OCD could be evolutionarily conserved. The fundamental phenotype involved in this process may represent lower-order deficits arising from alterations in neural development, which could influence higher-order cognitive processes in a “bottom-up” manner (Benzina et al., 2021; Poletti et al., 2022a,b). These findings are also consistent with research suggesting that deficits in lower-order sensory and affective processing may underline the cognitive and behavioral changes seen in patients with OCD (Cavedini et al., 2012; Martoni et al., 2015).

## Neurodevelopment and OCD in humans

The argument presented above would gain support if it were possible to demonstrate neurodevelopmental antecedents of OCD in humans. In this case, too, the available evidence suggests that at least some types of OCD have developmental antecedents. The evidence for these developmental alterations has been summarized in recent reviews (Poletti et al., 2022a) and includes altered cortical and white matter development in early-onset OCD (Li et al., 2021; Park et al., 2022), functional alterations in cortico-striato-thalamic circuits in childhood OCD (Huyser et al., 2009; Liu et al., 2016), a higher frequency of neurological soft signs in OCD patients with poor insight or comorbid tics (Karadag et al., 2011; Ekinci and Ekinci, 2020), subtle alterations in facial morphology in early-onset OCD (Wang et al., 2021), and associations between OCD and events that could alter brain development either pre- or perinatally (Vasconcelos et al., 2007) or in early childhood (Barzilay et al., 2019; Wislocki et al., 2022).

Specific phenotypes have also been linked to these developmental alterations. For example, patients with OCD show evidence of impaired olfaction, which is a marker of brain dysfunction of developmental origin (Crow et al., 2020). However, the most frequently replicated phenotype of possible developmental origin in OCD involves alterations in sensory processing, most specifically sensory over-sensitivity. This phenomenon, which is characterized by increased sensitivity and reactivity to sensory stimuli in various modalities, has been documented both in children and adolescents (Houghton et al., 2020) and adults (Dar et al., 2012; Isaacs et al., 2022) with OCD. Sensory over-sensitivity has also been associated with childhood ritualistic behavior (Dar et al., 2012). While early childhood rituals are common and are usually “outgrown” in later childhood and adolescence, they may persist and evolve into OCD in some cases; in these cases, they may represent a developmental precursor of the full syndrome of OCD (Leckman and Bloch, 2008; Evans et al., 2011). Like clinical OCD, these childhood forerunners are associated with alterations in functional connectivity between limbic, sensorimotor, striatal and thalamic brain regions (Sunol et al., 2021). These changes have been linked to alterations in genes linked to glutamatergic neurotransmission (Sunol et al., 2022), which is one of the key pathways disrupted in *SAPAP3* or *SPRED2* knock-out mice.

## Genetics, neurodevelopment and endophenotypes in OCD

An endophenotype is a heritable trait that can be measured in an objective manner, and which is present in individuals with a given psychiatric disorder, as well as their unaffected first-degree relatives, at rates significantly higher than in healthy controls or in the general population (Gottesman and Gould, 2003). It represents an “intermediate phenotype” that is genetically linked to the disorder in question and more amenable to study using biological methods. A number of candidate endophenotypes have been proposed for OCD (Vaghi, 2021). These include alterations in specific domains of cognition (Zartaloudi et al., 2019; Bora, 2020), structural abnormalities in specific brain regions such as the insula (Besiroglu et al., 2022), and altered patterns of functional activity within and between specific neural circuits involved in sensorimotor function, cognition and resting-state activity (Peng et al., 2021). Among these, cognitive endophenotypes have been the most frequently documented (Vaghi, 2021) and have been observed even in pediatric OCD (Abramovitch et al., 2021). Recent evidence suggests that the polygenic risk score, a measure of genetic vulnerability toward OCD, is significantly correlated with alterations in brain activity during the performance of cognitive tasks not just in patients with OCD and their unaffected relatives, but in healthy controls (Heinzel et al., 2021). This result suggests the possibility of a “continuum” of genetic vulnerability to OCD that could cause subtle deficits in higher-order cognitive functions, most probably through alterations in brain development and functional connectivity. Such a continuum has also been demonstrated in a genome-wide analysis of pediatric obsessive-compulsive disorder and traits (Burton et al., 2021). In the latter study, suggestive associations were identified for the genes GRID2 and PTPRD, which may be functionally linked to SAPAP3 (Pauls et al., 2014). As of now, there is no direct evidence linking this putative genetic continuum to abnormalities of sensory processing. However, a recent study of over 1,400 adolescents and adults, examining the entire spectrum of obsessive-compulsive phenomena, reported that sensory over-responsiveness was associated with this spectrum in a transdiagnostic manner (Moreno-Amador et al., 2023). Though requiring replication, this result suggests that the possibility of a genetically influenced neurodevelopmental vulnerability to symptoms across the OC spectrum may be linked to sensory over-sensitivity. This implies that the latter may be a useful endophenotype for OCD.

## Integrating bottom-up and top-down approaches in the study of clinical OCD

**Figure 1:**
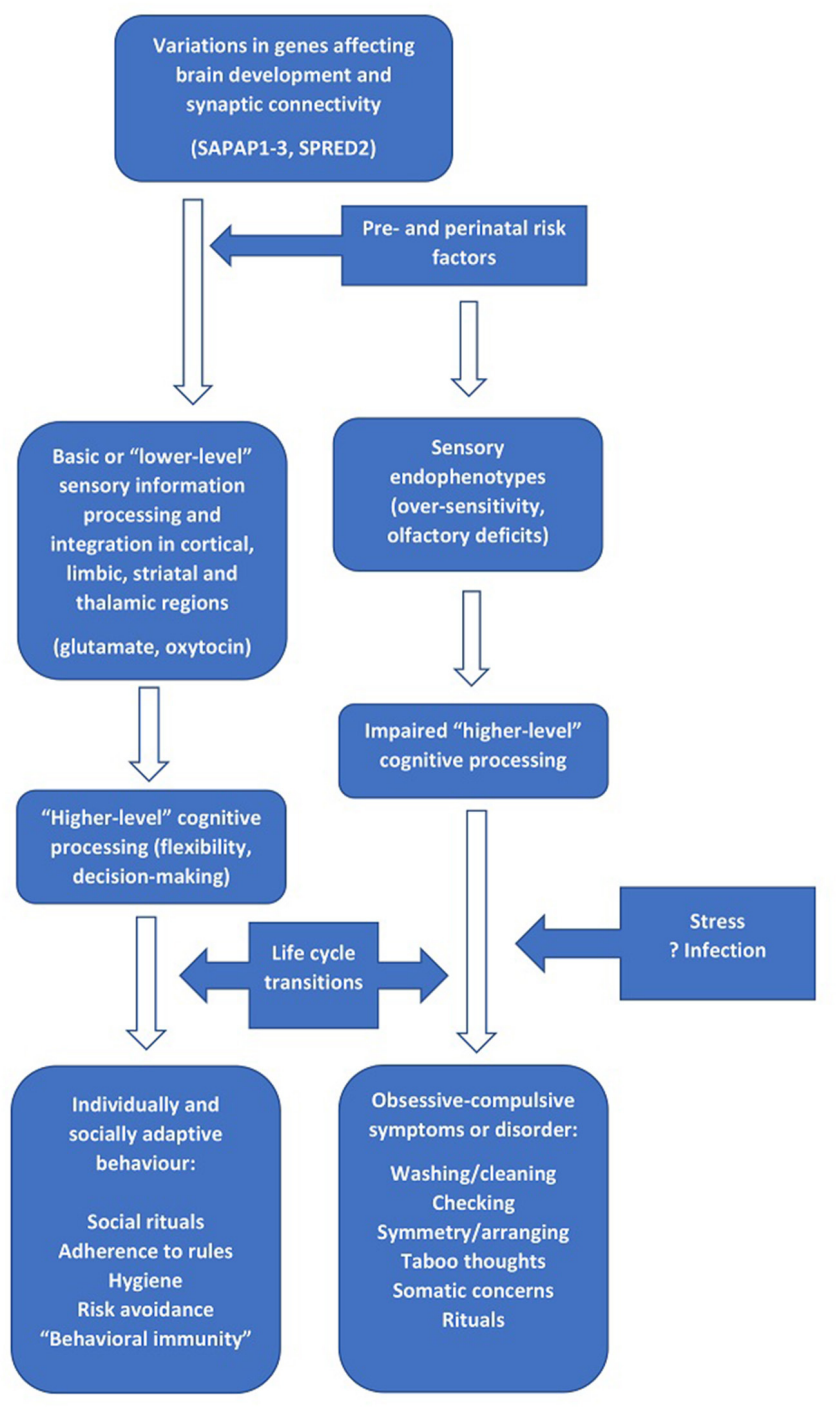
The relationship between genes involved in brain development and synaptic connectivity, “lower-” and “higher-order” information processing, and the development of obsessive-compulsive symptoms.

A tentative integration of the findings described above is presented in Figure 1, with the left-hand side of the figure indicating “normal” development and the right-hand side indicating the processes implicated in the pathogenesis of OCD. In this model, genetic factors (particularly those involved in neural development and synaptic scaffolding) interact with pre, peri- and post-natal exposures to cause structural and functional alterations in brain circuits involved in “lower-level” processes that are operational from early childhood, such as sensory processing and early social behavior (de Oliveira et al., 2021; Schiele et al., 2022). The available evidence suggests that neurotransmitters such as glutamate (Karthik et al., 2020; Auerbach et al., 2021) and oxytocin (Crucianelli et al., 2019; Bey et al., 2022) may be particularly involved in these processes as well as in OCD.

During the course of cognitive development in early life, alterations in these processes affects “higher-order” cognitive processes such as flexibility and decision-making capacities (Abramovitch et al., 2021). In children with no or minimal alterations to these circuits and processes, the result is transient childhood rituals and the subsequent development of appropriate rule- or ritual-based social and risk-avoidance behavior in later life. In those with more marked alterations in these processes, a transition to clinical OCD occurs (Poletti et al., 2022b). This may be more likely to happen at “critical” stages of the life cycle, or at any other period characterized by increased stress and a higher cognitive, affective or sensory load (Sousa-Lima et al., 2019; Imthon et al., 2020; Raposo-Lima and Morgado, 2020). Such periods include the transition from early to later childhood (Geller et al., 2001), the transition from adolescence to adulthood (Horwath and Weissman, 2000; Solmi et al., 2022), and pregnancy or childbirth in women (Starcevic et al., 2020). In other cases, infections or immune-inflammatory alterations may act as triggering factors (Gerentes et al., 2019). Finally, in those with intermediate alterations and/or lower levels of stress, subsyndromal OCD symptoms may occur and persist for a variable period (Fullana et al., 2009; Ramakrishnan et al., 2022).

## Conclusions

The model outlined above represents a tentative yet coherent approach to understanding the mechanisms through which evolutionarily conserved cellular and neurobiological processes could contribute to the development of OCD in humans. Much remains to be learned about the specific association of each process with OCD, their relationship to the different dimensions of OCD, and the opportunities they offer for early intervention, improved treatment, and the identification of specific endophenotypes such as sensory over-sensitivity (Fontenelle et al., 2022). While many of the conclusions presented here require verification, they could potentially deepen our understanding of OCD and its evolutionary and developmental roots.

## Author contributions

The sole author of this work was responsible for the conceptual framework, literature review, writing, editing, and proofreading of this paper.
